# Chest Pain Diagnosed as Acute Leukemia: Focus on Coagulation Abnormalities Rather Than White Blood Cell Count

**DOI:** 10.7759/cureus.35992

**Published:** 2023-03-10

**Authors:** Kyohei Maeno, Kasumi Satoh, Nobuhisa Hirasawa, Manabu Okuyama, Hajime Nakae

**Affiliations:** 1 Department of Emergency and Critical Care Medicine, Akita University Graduate School of Medicine, Akita, JPN

**Keywords:** coagulation abnormalities, white blood cell count, disseminated intravascular coagulation, acute myeloid leukemia, chest pain

## Abstract

Chest pain is an important symptom for emergency physicians. It is one of the most common causes for admission in emergency departments. Acute leukemia (AL) rarely causes chest symptoms due to ostalgia, and it is difficult to diagnose leukemia as the cause of chest pain. An 83-year-old woman with no history of trauma presented to the emergency department with a one-day history of severe chest pain. There were no abnormalities on electrocardiography, echocardiography, specific biomarkers for cardiac injury, or contrast computed tomography of the chest and pelvis. The white blood cell count was normal, but the patient had prominent thrombocytopenia with platelets of 68,000/µL, prothrombin time-international normalized ratio (PT-INR) of 1.2, activated partial thromboplastin time (APTT) of 25.3 s, and D-dimer of 73.55 µg/mL. Due to the holiday, blast cells could not be measured on the same day. The next day's examination revealed blast cells in the peripheral blood. The patient was admitted to the hematology department and discharged three months later. This case suggests the need to consider AL in chest pain associated with coagulation abnormalities and thrombocytopenia, regardless of the white blood cell count.

## Introduction

Acute leukemia (AL) is a hematologic malignancy resulting from mutations in bone marrow stem cells [1]. Acute myeloid leukemia (AML) is the most frequent type of acute leukemia in adults, accounting for roughly 80% of cases in adults [2]. AML presents with a variety of symptoms, such as malaise, gingival hemorrhage, and nasal hemorrhage. It causes bone pain in 4% of adult AL cases [3]. AL can cause chest pain if it occurs in the sternum or ribs [4,5]. As AL can rapidly become severe, emergency physicians should be aware of AL as a cause of chest pain.

Here, we present an atypical case of chest pain caused by AL in the emergency department. In this case, coagulation abnormalities provided clues to the AL.

## Case presentation

An 83-year-old woman presented to the emergency department (ED) with severe anterior chest pain that had persisted from the previous day. The patient complained of severe sternal pain. Pain was present even at rest and was enhanced by compression of the sternum. The vital signs were as follows: blood pressure, 144/82 mmHg; pulse rate, 75 beats/min; oxygen saturation, 94% on ambient air; respiratory rate, 14 breaths/min; and body temperature, 36.6℃. Electrocardiography showed sinus rhythm with no ST-T changes, and echocardiography showed no evidence of left ventricular regional wall motion abnormalities or valvular disease.

Laboratory tests showed no changes in the levels of serum creatine kinase muscle and brain isoenzyme (CK-MB) and troponin T. However, the complete blood count (CBC) showed thrombocytopenia and coagulation abnormalities were evident (Table 1, first column). The patient's Japanese Society of Emergency Medicine acute disseminated intravascular coagulation (DIC) score was four points. A diagnosis of DIC was established. However, the etiology remained unidentified. 

**Table 1:** The complete blood count (CBC) and coagulation in the first admission to the ED and the second admission, with respective reference range levels. ED: emergency department.

	First examination to ED	Second examination to ED	Reference range levels
White blood cell count	7000/μL	7800/µL	4000-10,000/µL
Neutrophil count	11%	21%	40-69%
Lymphocyte count	34%	15%	26-46%
Eosinophil count	0%	0%	0-5%
Basophil count	0%	1%	0-2%
Monocyte count	0%	1%	2-12%
Blast count	36%	55%	-
Hemoglobin	11.4 g/dL	10.9 g/dL	11.4-14.6 g/dL
Platelet count	6.8 × 10^4^/μL	1.9 × 10^4^/μL	15-33 × 10^4^/μL
Activated partial thromboplastin time (APTT)	25.3 s	26.0 s	20-40 s
Prothrombin time-international normalized ratio (PT-INR)	1.2	1.37	0.85-1.15
D-dimer	73.55 μg/mL	261.60 μg/mL	<1.0 μg/mL
Fibrinogen/fibrin degradation products (FDPs)	549.4 μg/mL	549.9 μg/mL	<5.0 μg/mL

**Figure 1:**
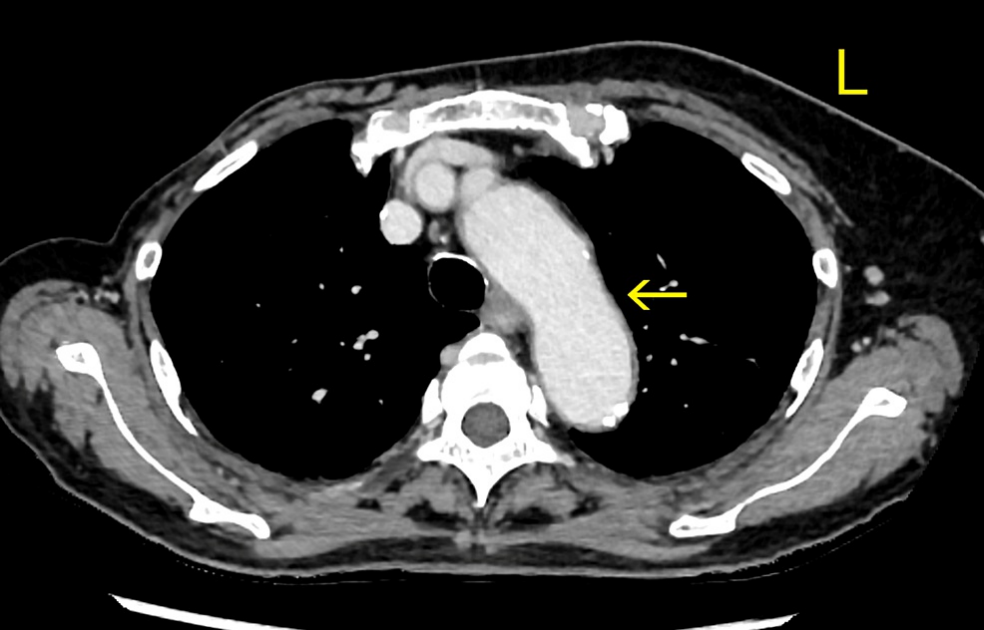
Computed tomography of the chest and pelvis in the first admission. The yellow arrow indicates the aorta; there was no evidence of acute pulmonary embolism and aortic dissection on CT. CT: computed tomography.

Contrast-enhanced computed tomography (CT) of the chest and pelvis showed no acute pulmonary embolism or aortic dissection (Figure 1).

The physician ruled out acute coronary syndrome, aortic dissection, and pulmonary embolism as the cause of chest pain. On the first day, acute and critical illnesses were ruled out, and the patient was sent home with a prescription for analgesics. Next day, the CBC revealed 55% of blast cells, and the coagulation abnormality developed significantly over one day (Table 1, second column). The patient was diagnosed with acute leukemia (AL) and concurrent disseminated intravascular coagulation (DIC).

**Figure 2:**
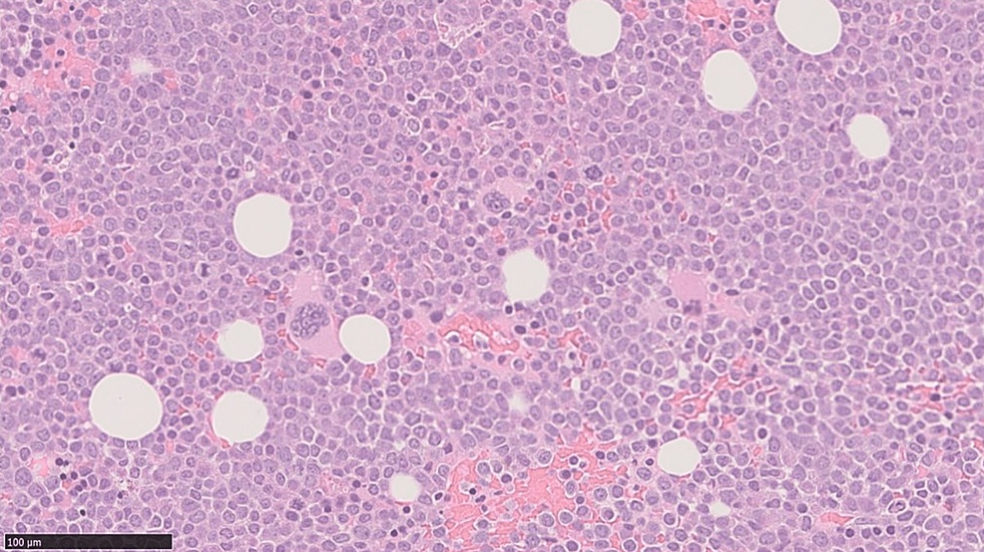
Bone marrow aspiration on admission.

The patient was admitted to the hematology department for further evaluation and treatment of AL. A bone marrow aspiration test (Figure 2) revealed that 84% of the blast cells accounted for a nucleated cell count of 578,000/µL. The patient was initially diagnosed with acute promyelocytic leukemia (APL). All-trans retinoic acid and arsenic trioxide were started; however, the treatments were discontinued within three days due to pulmonary edema. On the 12th day of the illness, promyelocytic leukemia-retinoic acid receptor alpha (PML-RARα) testing was conducted and returned negative results, leading to a revision of the diagnosis to acute myeloid leukemia (AML). The patient completed two courses of chemotherapy (venetoclax and azacitidine) and was discharged three months after admission.

## Discussion

This case highlights the importance of considering AL as a cause of acute-onset chest pain in the ED. AL should be investigated regardless of the white blood cell count, especially in cases of coexisting coagulation abnormalities and thrombocytopenia.　　

Chest pain in the ED can often be a cause of fatal disease. For example, a myocardial infarction occurs in approximately 15% of patients with chest pain in the ED [6]. Chest pain with AL is reportedly caused by increased intramedullary pressure and bone destruction; thus, chest pain is often expressed as musculoskeletal pain [7]. Since diseases causing non-cardiac chest pain are rarely fatal [8], emergency physicians tend to underestimate the significance of musculoskeletal pain. Ostalgia due to AL is more common in children (14%), but 4% of adults also present with bone pain [3]. Bone pain due to AL is more common in the fibula and tibia, spine, and femur, and less frequently in the sternum [9]. Several previous reports of AL with chest pain are available [4,5]. It is difficult to diagnose AL as a cause of chest pain in the ED at first glance because AL with chest pain occurs infrequently. However, it is essential for emergency physicians to diagnose AL because it can trigger serious infections and hemorrhage.

AL can present with elevated or normal or low white cell count. In the early stages of AL, proliferating leukocytes remain in the bone marrow, and peripheral leukocyte counts decrease because normal hematopoiesis is impaired. As the disease progresses, leukemic cells migrate into the peripheral blood, and the leukocyte count increases. Hu et al. reported that 15.8% of patients aged <60 years and 9.8% of patients aged >60 years were within the threshold for white blood cell count at diagnosis [10]. In a study of 986 elderly patients with AML, low leukocyte counts and a tendency toward a low percentage of myeloblasts were observed [11]. Therefore, more attention should be paid to elderly patients.

When leukocytes in the peripheral blood are normal, coagulopathy may be a clue to the diagnosis of AL. Acute promyelocytic leukemia (APL) has a high frequency of coagulopathy, with a DIC complication rate of 78.0% compared to 31.6% for AML excluding APL [12]. Although this case was diagnosed as AML cuplike, a report of the same type of cases showed a DIC complication rate of 57.1%, suggesting that this type of patient may be prone to DIC as well as APL [13]. In a report on all types of AL, DIC was found in 31.58% of patients [14]. In all types of AL, >30% of patients present with DIC, and coagulation abnormalities are likely to trigger a diagnosis of AL. Similarly to our case, Sakata et al. reported a case in which AL was the cause of generalized pain in the ED, and the white blood cell count was normal, but D-dimer elevation and thrombocytopenia were evident, leading to a diagnosis of AL [15].

## Conclusions

When bone pain due to AL extends to the ribs or sternum, it manifests or presents as chest pain. Chest pain is a common complaint in the ED, and emergency physicians should be skilled in chest pain management. It is not easy to recall AL with chest pain in the ED, as in this case. If chest pain is complicated by thrombocytopenia or elevated FDP and D-dimer levels, AL should be considered. Regardless of the peripheral blood leukocyte count, leukocyte fractionation of the peripheral blood should be confirmed, and a bone marrow examination should be performed.
